# Clinical Practice Patterns in the Physiotherapy Management of Tension-Type Headache Among Spanish Physiotherapists

**DOI:** 10.3390/jcm15082896

**Published:** 2026-04-10

**Authors:** Ana Bravo-Vazquez, Elena De-La-Barrera-Aranda, Ernesto Anarte-Lazo, Cleofas Rodriguez-Blanco, Carlos Bernal-Utrera

**Affiliations:** 1 Doctoral Program in Health Sciences, University of Seville, 41009 Seville, Spain; ana.bravo@fisiosurid.es; 2 Physiotherapy Department, Faculty of Nursing, Physiotherapy and Podiatry, University of Seville, 41009 Seville, Spain; cleofas@us.es (C.R.-B.); cbutrera@us.es (C.B.-U.); 3 Fisiosur I+D, Research Institute, 04630 Garrucha, Spain; en2baare@uco.es; 4 Morphological and Socio-Health Sciences Department, University of Cordoba, 14071 Cordoba, Spain; 5 Faculty of Health, UNIE University, 28015 Madrid, Spain

**Keywords:** tension-type headache, physiotherapy, manual therapy, therapeutic exercise, clinical practice

## Abstract

**Background**: Tension-type headache (TTH) is the most prevalent primary headache disorder worldwide and represents a major source of disability related to chronic pain. Despite its high prevalence, uncertainty remains regarding optimal conservative management strategies, and limited evidence is available on how physiotherapists apply existing recommendations in routine clinical practice. **Objective**: The objective was to explore physiotherapists’ perceptions, clinical experiences, and treatment strategies in the management of tension-type headache, with particular emphasis on commonly used interventions, clinical decision-making, and characteristics of physiotherapy care. **Methods**: A cross-sectional survey study was conducted using a self-administered online survey developed in accordance with the CHERRIES guidelines. One hundred Spanish physiotherapists with clinical experience in treating patients with TTH participated. Quantitative data were analyzed descriptively, while open-ended responses were examined using inductive thematic analysis following the framework proposed by Braun and Clarke. **Results**: Manual therapy was the most frequently reported intervention (96%), followed by therapeutic exercise (61%) and invasive techniques, primarily dry needling (48%). The suboccipital and upper cervical regions were consistently identified as primary therapeutic targets, reflecting a predominant craniocervical treatment focus. Most respondents reported individualized treatment plans, typically delivered in weekly sessions lasting 45–60 min, with expected clinical improvement within 4–6 weeks. Pain education strategies were reported infrequently. Considerable variability was observed in the selection and combination of therapeutic techniques. **Conclusions**: Physiotherapists managing tension-type headache commonly adopt a multimodal approach, largely centered on manual and tissue-focused interventions. Although many reported practices are aligned with current evidence, the substantial heterogeneity observed and the limited integration of biopsychosocial strategies highlight the need for consensus-based guidelines and further research addressing real-world clinical effectiveness.

## 1. Introduction

Tension-type headache (TTH) is the most prevalent primary headache disorder worldwide, affecting approximately 26–38% of the population, making it one of the leading causes of disability and socio-economic burden associated with chronic pain disorders [1]. Its impact goes beyond the physical sphere, significantly affecting quality of life, daily activities, and work productivity, as well as promoting the overuse of analgesics [2]. Despite its high prevalence, medical consultation rates for TTH remain low, possibly due to limited information on effective conservative treatments or previous negative experiences in healthcare [3].

Clinically, TTH is characterized by bilateral pain with a pressing or tightening quality, generally of mild to moderate intensity and variable duration, ranging from minutes to several days. It typically does not worsen with routine physical activity and is less commonly accompanied by nausea, photophobia, or phonophobia. However, clinical overlap with other headache types may complicate diagnostic accuracy and classification [4,5,6]. Although TTH is highly prevalent, its underlying etiology and pathophysiological mechanisms are still not fully understood. Proposed mechanisms include peripheral nociception originating from cervical musculoskeletal structures and convergence of cervical and trigeminal afferents within the trigeminocervical nucleus, as well as altered pain modulation and features consistent with central sensitization, especially in chronic forms of the disorder [7,8,9].

Management of TTH typically incorporates both pharmacological and non-pharmacological approaches. Within the latter, physiotherapy has demonstrated potential benefits in reducing pain intensity and improving function [10,11,12]. Nonetheless, the evidence remains heterogeneous, partly due to variability in treatment protocols, the selection and combination of techniques, the dosage and frequency of interventions, and the outcome measures used across studies [13,14]. Furthermore, many clinical trials have focused exclusively on quantitative outcomes, offering limited insight into physiotherapists’ clinical reasoning processes or the decision-making strategies underpinning their therapeutic choices [11].

Physiotherapists play a central role in the multidisciplinary management of TTH, assessing cervical and cranial musculoskeletal dysfunctions, designing individualized intervention plans, and guiding patients throughout their clinical management [15]. However, despite their key involvement, there is limited literature describing how physiotherapists approach TTH in real-world practice, which techniques they use most frequently, how they prioritize different therapeutic tools, and which interventions they perceive as most effective [16].

Although systematic reviews and randomized controlled trials have evaluated the effectiveness of physiotherapy interventions for TTH, these studies generally focus on clinical outcomes and do not explore the perspectives and experiences of the professionals responsible for delivering these treatments [17]. This gap restricts a comprehensive understanding of how evidence-based recommendations are translated into clinical practice and how physiotherapists adapt interventions to individual patient needs [18].

Several studies have explored physiotherapists’ clinical practice patterns in the management of musculoskeletal pain in other countries, including low back pain, knee osteoarthritis, whiplash-associated disorders, and shoulder pain [19,20,21,22,23]. These surveys reveal both consistencies and variability in clinical practices, reflecting differences in training, clinical settings, and guideline implementation. Integrating this international evidence highlights the gap in knowledge regarding tension-type headache management and underscores the relevance of examining clinical practices in Spain.

Therefore, the primary objective of this study was to explore the clinical practice patterns of Spanish physiotherapists in the management of TTH. In particular, the study aimed to investigate which therapeutic interventions are most commonly used, physiotherapists’ perceptions regarding their application, and the characteristics of the applied treatment protocols. By means of an online survey, this research sought to identify current physiotherapy practices, decision-making criteria, and perceived effectiveness of commonly used interventions. The findings of this study are intended to contribute to a deeper understanding of the physiotherapeutic approach to TTH and to support the development of optimized, evidence-informed treatment strategies.

## 2. Materials and Methods

### 2.1. Study Design

A cross-sectional mixed-methods survey study was conducted using a self-administered online survey. The methodology was designed and reported following the Checklist for Reporting Results of Internet E-Surveys (CHERRIES), translated into Spanish [24]. This mixed-methods approach allowed us to explore both the frequency and patterns of therapeutic interventions and the perceptions, experiences, and rationale reported by physiotherapists in their clinical practice regarding the physiotherapeutic management of TTH.

### 2.2. Participants and Recruitment

Participants were licensed physiotherapists currently practicing clinically and/or conducting research related to the treatment of patients with TTH. A combination of convenience sampling and snowball sampling was used to facilitate the recruitment of physiotherapists with specific experience in headache management and cervical musculoskeletal disorders. The study was designed and reported following the CHERRIES guidelines [19], and measures were taken to ensure participant anonymity, prevent multiple responses, and provide transparent reporting of survey administration.

The survey, with an approximate duration of 10 min, also included questions to confirm participants’ experience in the physiotherapeutic management of TTH. Physiotherapists were recruited through multiple overlapping channels: (a) web and professional network searches; (b) review of scientific publications and books in physiotherapy and headache care; (c) prior knowledge of the research team; (d) recommendations from experts in neuromusculoskeletal physiotherapy; and (e) contact with scientific societies and professional physiotherapy associations.

Participation was entirely voluntary and anonymous. Initially, the survey was sent privately by email to 70 physiotherapists known to the research team. Subsequently, the survey was also distributed via LinkedIn, professional networks, and third-party contacts using a snowball sampling approach. Due to this combination of convenience and snowball sampling, the exact number of physiotherapists who ultimately received the invitation could not be determined, and a formal response rate could therefore not be calculated.

The survey was available from 14 October to 14 November 2025. Informed consent was obtained electronically before accessing the questionnaire, with participants confirming agreement via a checkbox. Only those who provided consent could proceed with the survey.

The survey was closed after 100 responses were obtained. Although a formal sample size calculation was not conducted due to the exploratory nature of the study, the sample size was considered adequate for the study objectives. In descriptive survey-based research, there is no universally established sample size, as its appropriateness depends on the type of analysis and feasibility of recruitment. Methodological studies suggest that samples in the range of approximately 50–120 participants are common in observational health research and allow for stable descriptive estimates comparable with existing literature [25]. Additionally, this number was sufficient to capture a diverse range of perspectives and achieve data saturation in the qualitative analysis of open-ended responses.

### 2.3. Questionnaire Development

The questionnaire was developed using Google Forms (Google LLC, Mountain View, CA, USA) by a team of physiotherapists with clinical and research experience in musculoskeletal pain and cranio-cervical disorders. Its design was based on previous literature on physiotherapy for TTH [16,17], and reference clinical manuals in the field [26], complemented by consultation with three expert physiotherapists in headache and cervical pain management to ensure content validity.

Items were drafted to cover the main domains of physiotherapeutic management: clinical practice and most frequently used techniques, perceptions of priority intervention techniques, and intervention protocols. A pilot test was conducted with five physiotherapists who were not included in the final sample to assess clarity, comprehensibility, and face validity. Based on their feedback, several items were reworded to improve readability and relevance.

While formal psychometric evaluation (e.g., internal consistency, construct validity, test–retest reliability, or factor analysis) was not conducted, these steps ensured that the questionnaire had content validity, face validity, and practical relevance, in line with the exploratory nature of the study.

The final questionnaire included both closed- and open-ended questions, organized into five sections, as shown in Table 1. Representative examples of each domain are provided at the end of each column. The complete survey is available in the Supplementary Materials (File S1).

### 2.4. Ethical Considerations and Informed Consent

The study involved an anonymous survey of licensed physiotherapists and did not collect personal health information or involve clinical interventions. According to standard practice for survey research involving professionals, formal ethical approval was not required.

Before accessing the questionnaire, participants received an information sheet explaining the study objectives, data protection measures, confidentiality of information, and the voluntary nature of participation. Informed consent was obtained by selecting a checkbox indicating acceptance. Only physiotherapists who provided consent were able to access the survey. The study was conducted in accordance with the ethical principles established in the Declaration of Helsinki.

### 2.5. Data Protection and Prevention of Duplicate Responses

To ensure data integrity, responses were manually reviewed for potential duplicates. Duplicate detection was performed by comparing submission times, sociodemographic information, and professional profiles. No IP addresses or personally identifiable information were collected. All data were securely stored in a password-protected institutional account, accessible only to the research team. These procedures were implemented in line with CHERRIES recommendations to ensure anonymity, prevent multiple responses, and maintain transparency in online survey reporting.

### 2.6. Data Analysis

Quantitative variables were analyzed using descriptive statistics (means, standard deviations, frequencies, and percentages) with SPSS software (version 31.0.0 for macOS).

These analyses provided an overview of the prevalence and patterns of therapeutic interventions among Spanish physiotherapists. Given the exploratory nature of this study and the sample size, the analysis was limited to descriptive statistics.

Complementarily, open-ended responses were analyzed using inductive thematic analysis following the phases proposed by Braun and Clarke [27]: familiarization with the data, generation of initial codes, searching for themes, reviewing themes, defining and naming themes, and reporting findings. An audit trail was maintained documenting coding decisions and theme development. Representative illustrative quotations for each theme are provided in Table 1.

Coding was conducted collaboratively through a series of working meetings among the authors, during which initial codes were developed inductively, discussed, and refined until consensus was reached. Data saturation was continuously monitored, and no new codes or themes emerged during the final stages, indicating that the sample size (*n* = 100) was sufficient to capture the range of perspectives.

This qualitative analysis allowed exploration of physiotherapists’ perceptions and experiences regarding the management of TTH, complementing the quantitative findings and providing a more comprehensive understanding of clinical practice patterns.

A detailed audit trail documenting coding decisions and theme development was maintained to ensure transparency. Data saturation of the open-ended responses was continuously monitored. No new codes or themes emerged during the final stages of coding, and the responses from the last participants confirmed thematic saturation, indicating that the sample size (*n* = 100) was sufficient to capture the range of perspectives among participants [28].

## 3. Results

A large number of physiotherapists were invited to participate in our survey via email, which included a link to the Google Forms questionnaire. Recruitment was conducted using our predefined algorithm to ensure that participants had relevant experience in the management of the condition under study. Although many invitations were sent to reach a broad sample, the survey was closed once a total of 100 responses had been received. This approach allowed us to obtain a sufficient and manageable sample size for analysis.

### 3.1. Sociodemographic Characteristics and Professional Information

The sample consisted of 100 physiotherapists with an equal gender distribution and a mean age of 36 years (SD = 9.27), indicating a relatively young professional group but one with substantial experience, as nearly half reported more than 10 years of practice. Regarding academic background, postgraduate qualifications were predominant, with 44% holding a master’s degree and 16% having completed a doctorate, a proportion consistent with the advanced training and time commitment required for doctoral studies. Most participants worked in private clinical practice (66.8%), reflecting a predominantly clinical and patient-oriented professional profile, while smaller proportions were involved in university teaching, research, or public institutions. Concerning exposure to patients with this condition, 73% treated fewer than 20 cases per month, suggesting that the pathology is relatively infrequent in typical clinical practice, whereas only 3% reported high case volumes, likely due to specialized training or high service demand. Overall, the sample represents a well-educated and experienced physiotherapy workforce primarily engaged in private practice, with variable levels of clinical exposure to the condition under study. Detailed sociodemographic and professional characteristics of the participants are presented in Table 2.

### 3.2. Treatment Techniques Routinely Used

**Figure 1:**
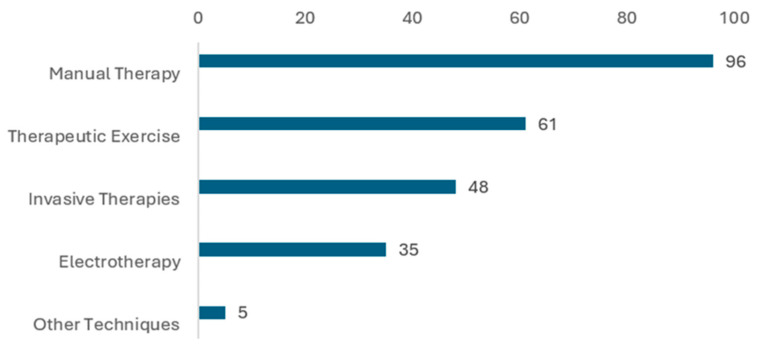
Treatment techniques routinely used.

Manual Therapy is the clearly predominant technique, used by 96% of physiotherapists; Therapeutic Exercise ranks second (61%), Invasive Therapies show a considerable presence (48%), Electrotherapy is used by just over one third of practitioners (35%), and other techniques (5%) show low representation. Treatment techniques routinely used are summarized in Figure 1.

### 3.3. Most Commonly Used Modalities and Areas Most Frequently Treated with Each Technique

The survey identified a broad array of therapeutic modalities employed by physiotherapists for the management of TTH. Tables S1–S5 in the Supplementary Material present the frequency of each modality. In summary, suboccipital muscle inhibition, cervical joint mobilization, and trapezius massage were the most reported manual techniques. Exercise-based interventions commonly included cervical mobility, scapular strengthening, and postural control. Among invasive procedures, dry needling targeting the upper trapezius and suboccipital muscles was most frequent. Electrotherapy and adjunctive methods (e.g., pain education, kinesiology taping) were used less consistently.

#### 3.3.1. Manual Therapy

**Figure 2:**
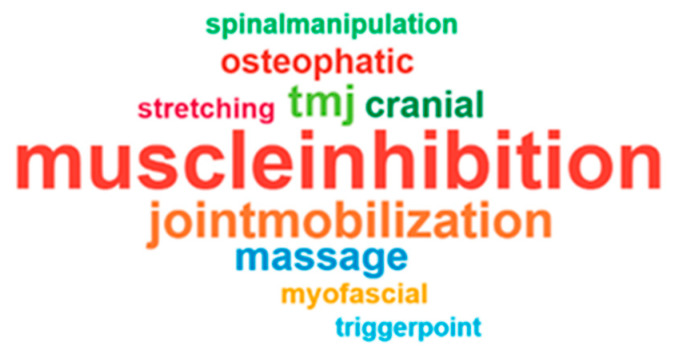
Word-cloud of the most frequently reported treatment modalities. The size of each term is proportional to the percentage of respondents who reported using that modality in their clinical practice.

Muscle inhibition at the suboccipital region was reported by 46 participants, representing the highest frequency. Myofascial release, stretching/relaxation of cervical and paravertebral muscles, and high-cervical joint mobilizations (C0–C3) were also frequently cited. Treatment of the temporomandibular joint (TMJ), intraoral techniques, cranial/craniosacral therapy, and osteopathic manipulations were mentioned by a subset of respondents, indicating a varied manual therapy repertoire (see Table S1). Figure 2 shows a word-cloud representation of the most frequently mentioned treatment modalities, where the size of each term reflects the percentage of respondents who reported using that modality.

#### 3.3.2. Therapeutic Exercise

**Figure 3:**
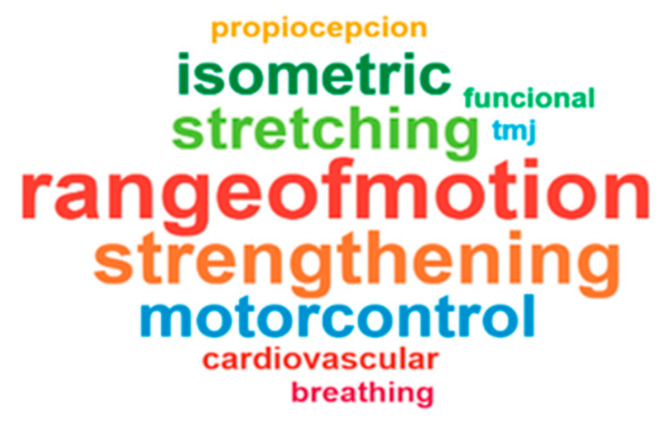
Word-cloud of treatment modalities most frequently reported by surveyed physiotherapists; font size reflects the percentage of respondents citing each modality.

Cervical range-of-motion exercises were cited 22 times; scapular/shoulder and thoracic mobilizations, as well as TMJ/cranio-cervical mobilizations, were reported less frequently. Strengthening/endurance exercises for cervical and scapular/shoulder regions appeared in 15 and 10 case reports, respectively. Isometric cervical exercises were noted in 19 responses, and isometric work for shoulder muscles in 5. Stretching was documented in 15 cervical-focused cases and fewer for shoulder or thoracic muscles. Motor control/postural reeducation for cervical musculature was frequent, whereas proprioceptive training was uncommon. Additional modalities (aerobic exercise, diaphragmatic breathing, functional upper-body training, TMJ/ocular exercises, mind–body practices, neurodynamic) appeared with lower frequency (see Table S2). Figure 3 displays a word-cloud illustrating the most frequently mentioned treatment modalities, with the size of each term proportional to the percentage of respondents reporting its use.

#### 3.3.3. Invasive Therapies

Dry needling was the leading invasive technique, especially for trapezius (upper, middle, general; 22 mentions) and suboccipital muscles (14). Other targets included masticatory, levator scapulae, sternocleidomastoid and splenius/semispinalis muscles. Percutaneous electrical nerve stimulation (PENS) was used by 13 respondents for general musculoskeletal conditions, by 9 for Arnold’s nerve/occipital nerve and by 4 for trigeminal nerve involvement; spinal/neuroaxis applications were less frequent. Acupuncture was rarely reported (1 mention). See Table S3 for details.

#### 3.3.4. Electrotherapy and Physical Agents

**Figure 4:**
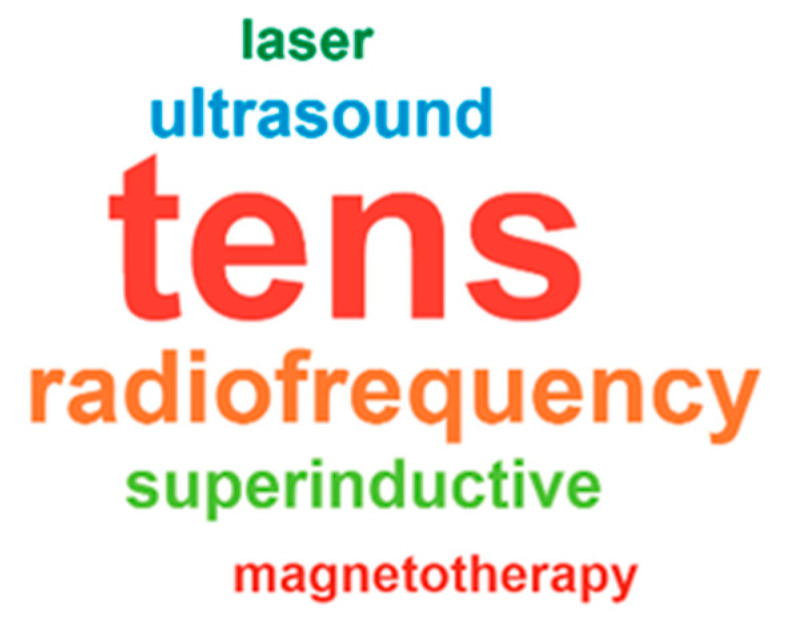
Word-cloud summarising the relative frequency of treatment modalities used in clinical practice; larger words correspond to modalities more frequently cited by participants.

TENS was the most common electrotherapy modality, especially over cervical (15) and trapezius/scapular (12) regions, with less frequent use over occipital/nerve or TMJ/cranio-cervical areas. Superinductive therapy/radiofrequency diathermy was applied in cervical, thoracic, and suboccipital zones (12 mentions), with fewer cervical/thoracic applications (4). Ultrasound, laser/inductive therapy, and magnetotherapy were rarely used (4, 2 and 1 mentions, respectively), and transcranial stimulation, microwave therapy or diamagnetic pump usage were minimal (each 1 mention). Table S4 summarizes these data. The word-cloud in Figure 4 reflects the relative frequency of the most cited treatment modalities, where larger font size corresponds to higher percentages of clinicians reporting each option.

#### 3.3.5. Other Techniques

Pain education was cited by 8 respondents, kinesiology taping (cervical, paravertebral, trapezius) by 6, while cupping and cross-tapping were each mentioned once. These supplementary techniques were used less frequently and likely served as adjuncts in multimodal treatment plans (see Table S5).

### 3.4. Techniques and Treatment Areas by Order of Preference

The three choices (1st, 2nd, and 3rd preferred technique and treatment area) reveal a clear pattern: the craniocervical region is the primary therapeutic target (almost 70% of all responses). The suboccipital area is consistently the highest initial priority, the upper cervical region (C0–C3) appears frequently as the second and third choice, and the trapezius, sternocleidomastoid (SCM), and deep cervical musculature show an increasing trend in the second and third selections. Altogether, this forms a characteristic “therapeutic triangle” composed of the Suboccipital-Upper Cervical-Trapezius/SCM regions. This pattern is also reflected in participants’ responses, for example: “I usually start with suboccipital inhibition techniques, as they tend to provide quick symptom relief,” and “My approach mainly involves manual therapy focused on the cervical spine and suboccipital region.”

Regarding the progression of techniques, the first choice is dominated by direct and intensive methods, such as suboccipital inhibition, mobilization and dry needling. For the second choice, modulatory and complementary techniques emerge, including massage, exercise, neuromodulation, and myofascial approaches. In the third choice, treatments such as therapeutic exercise, massage, and indirect or global techniques become predominant. This progressive approach is illustrated by responses such as: “I typically begin with manual techniques and then introduce exercise depending on patient response.” Table S6 shows the treatment techniques and target areas ranked by preference among the surveyed physiotherapists.

### 3.5. Characteristics of Physiotherapeutic Management in TTH

The data reveals a homogeneous profile in the physiotherapeutic approach to TTH, characterized by individualized interventions, moderate treatment duration, and a stable weekly frequency. Most physiotherapists place the expected clinical progression between 4 and 6 weeks (42%), followed closely by 1 to 3 weeks (40%), indicating that the majority of patients experience meaningful improvement within fewer than six weeks. A smaller proportion requires longer treatment durations (7–10 weeks: 10%; >10 weeks: 1%). Additionally, 7% reported a duration “depending on the case,” reflecting variability associated with individual factors such as chronicity, stress, or musculoskeletal comorbidities.

Regarding session frequency, the most common model is one session per week (51%), suggesting a strategy based on regular but spaced therapeutic stimuli. Twenty-five percent opt for two sessions per week, likely used in initial phases or more symptomatic presentations. The “depending on progression” option (21%) shows that a considerable proportion adjusts frequency based on clinical response. Very low frequencies (monthly) or very high frequencies (≥3/week) are exceptional and reserved for specific cases.

Standard practice centers around 45- to 60-min sessions (51%), followed by 30- to 45-min sessions (42%). Very brief (2%) or very long (5%) sessions are uncommon, likely related to contextual constraints or particularly complex cases.

The predominant trend is treatment individualization (83%). Seventeen percent combine structured protocols with customized adaptations, and no physiotherapist follows a fixed protocol.

Overall, these findings reflect a clinical practice oriented toward individualization, supported by a biopsychosocial framework and the need to combine multiple therapeutic modalities within a flexible structure.

## 4. Discussion

The present study provides a detailed and up-to-date overview of the clinical practices used by Spanish physiotherapists in the management of TTH, offering relevant information on both the professional profile of the participants and the therapeutic strategies most commonly applied in daily practice. The findings allow the identification of reported patterns in the musculoskeletal approach to TTH while also revealing substantial heterogeneity, which is indicative of the lack of consensus regarding optimal intervention protocols.

Regarding therapeutic trends, the most homogeneous finding was the predominance of manual therapy, used by 96% of physiotherapists, followed by therapeutic exercise (61%). Techniques targeting the suboccipital and upper cervical region were most frequently selected as first-line interventions. These included suboccipital muscle inhibition, a highly specific manual technique applied locally to reduce suboccipital muscle tone, as well as joint mobilisations and myofascial techniques, which represent broader manual therapy approaches aimed at addressing articular, muscular and fascial tissue restrictions across the cervical region. This therapeutic pattern is consistent with existing literature identifying C0–C3 dysfunction, suboccipital muscle hypertonicity and alterations in cervical motor control as key factors in the pathophysiology of TTH [29,30,31,32]. Previous systematic reviews have reported improvements in mobility, pain intensity and peripheral sensitivity when manual therapy and exercise are combined [15,33,34].

A notable finding is the widespread use of invasive therapies (48%), particularly dry needling. This proportion appears higher than that reported in studies conducted in Anglo-Saxon contexts, which may reflect differences in training frameworks and clinical traditions, including the strong presence of invasive myofascial techniques in Spain [35,36]. Although current evidence supports the short-term effectiveness of dry needling for pain modulation, its widespread use warrants consideration of factors such as cost-effectiveness, safety, practitioner training and appropriate risk management [37,38,39].

Electrotherapy was reported less frequently, which is broadly consistent with recommendations in current clinical guidelines. However, certain modalities with emerging evidence of effectiveness, such as diathermy or percutaneous neuromodulation, may be underutilised due to economic and logistical constraints, including equipment costs [40,41]. In contrast, more accessible modalities such as TENS continue to be commonly used, likely due to their low cost and ease of application [42].

Another relevant finding is the limited reported use of pain education strategies, despite strong evidence supporting their role in the management of pain conditions [43,44]. Several factors may potentially contribute to this pattern. First, physiotherapists may have variable or insufficient formal training in pain neuroscience, which can limit confidence in delivering neurophysiologically informed education. Second, time constraints during routine clinical sessions may restrict opportunities for structured patient education. Third, cultural and educational norms within physiotherapy practice may continue to prioritize hands-on, tissue-focused interventions over fully integrated biopsychosocial approaches [41].

Pain education, including the explanation of neurophysiological mechanisms, self-management strategies, and coping skills, has been shown to improve patient understanding, reduce fear-avoidance behaviors, and enhance adherence to active interventions [43,44]. Therefore, its limited use may represent a missed opportunity to optimize long-term outcomes. However, it is also important to recognize that other factors such as stress, sleep disturbances, and maladaptive coping strategies may influence symptom persistence and disability and often require additional interventions beyond pain education alone [44,45].

Overall, these findings suggest that further efforts are needed to promote the integration of pain neuroscience education within routine physiotherapy practice. Beyond educational and biopsychosocial strategies, other emerging therapeutic approaches targeting cranial and autonomic neural pathways should also be acknowledged. In particular, interventions aimed at modulating the sphenopalatine ganglion have been explored in the management of headache and facial pain conditions [46,47]. These include minimally invasive or non-invasive transnasal techniques involving the application of topical local anesthetics near the sphenopalatine foramen. Experimental evidence suggests that anesthetic agents applied in this region may diffuse into the pterygopalatine fossa and reach the ganglion, supporting the anatomical and physiological feasibility of this approach [48]. However, despite this mechanistic plausibility, clinical evidence remains limited and heterogeneous, and further research is needed to determine its effectiveness and potential role within multimodal management strategies.

Analysis of the techniques selected as first, second and third choices reveals a structured and staged intervention model as reported by respondents. The first phase targets the suboccipital and upper cervical region using specific manual techniques; the second incorporates interventions on the trapezius, SCM, masticatory muscles, TMJ and the greater occipital nerve, in line with trigeminocervical convergence; and the third phase emphasises therapeutic exercise, motor control, breathing and global strategies. This progression appears to be consistent with international guidelines and recent reviews advocating the combination of manual therapy and exercise as core components of TTH management, complemented by neurophysiological techniques [11,49].

Overall, the findings suggest that, according to respondents, manual techniques and dry needling are commonly employed and may offer short-term benefits; therapeutic exercise appears to play a key role in longer-term functional improvement, sensory modulation and relapse prevention [50].

The treatment patterns observed, 45-to-60-min sessions, weekly frequency and programmes of 1–6 sessions, are consistent with common musculoskeletal protocols and with evidence indicating that four to six sessions may be sufficient to achieve clinically meaningful improvements in pain and cervical mobility [51]. The small proportion of prolonged cases (>10 sessions or >10 weeks) may reflect more complex clinical presentations, potentially involving comorbidities, central sensitisation or adherence-related factors. The proportion of respondents reporting variable treatment frequency further highlights the importance of individualising interventions according to clinical evolution [52].

### 4.1. Comparison Between Clinical Practice and Available Evidence

The comparison between the responses obtained in the present survey and the findings derived from two recently published scoping reviews, one focused on the clinical characteristics of manual therapy interventions and the other specifically addressing dry needling in TTH [13,14], allows the routine clinical practice of Spanish physiotherapists to be interpreted in light of the available experimental evidence. Both reviews aimed to systematically map the types of techniques used, the anatomical structures targeted, treatment dosage, degree of individualization, diagnostic criteria, and outcome measures reported in randomized controlled trials, identifying areas of convergence as well as important gaps affecting clinical translation.

Overall, the survey findings show a substantial alignment with the evidence in terms of the central role of manual therapy in TTH management, particularly through techniques targeting the suboccipital and upper cervical regions and myofascial tissues. This consistency may support the application in clinical practice of therapeutic approaches grounded in established pathophysiological models, such as trigeminocervical convergence and cervical muscle hypertonicity.

At the same time, both the survey and the scoping reviews reveal considerable heterogeneity in the selection of techniques, target structures, treatment dosage, and therapeutic sequencing. This variability, evident in both research settings and daily practice, reflects the lack of standardized protocols and the ongoing difficulty in formulating clear, reproducible clinical recommendations. While most randomized controlled trials relied on standardized and minimally individualized protocols, the surveyed physiotherapists described a more progressive and adaptable approach, in which interventions are expanded according to clinical evolution and increasingly incorporate therapeutic exercise and more global strategies [13,14]. This contrast suggests that routine clinical practice may offer greater ecological validity, while also highlighting the need for research designs that better capture the complexity and variability of real-world TTH presentations.

With regard to dry needling, the survey indicates a broader clinical adoption of this technique than is currently represented in the randomized trials. The scoping review focusing on dry needling reports beneficial effects on headache intensity and frequency, as well as improvements in health-related quality of life, alongside a generally favorable safety profile. Nevertheless, it also highlights a limited number of studies and marked heterogeneity in target muscle selection, most commonly the upper trapezius and temporalis muscles, as well as in diagnostic criteria for myofascial trigger points, needling techniques, and treatment dosage. In addition, the limited differentiation between episodic and chronic TTH across studies further constrains the applicability of the findings. Taken together, these observations suggest that dry needling has been incorporated into clinical practice more rapidly than standardized, high-quality evidence has been generated to guide its optimal use, underscoring the importance of rigorous clinical reasoning, more objective diagnostic criteria, and adequate training in anatomy and risk management.

Overall, this comparative analysis represents one of the key contributions of the present study, demonstrating that physiotherapists’ reported practices reflect both the strengths and the limitations identified in scientific literature. These results support the need to move toward more standardized, individualized, and mechanism-informed protocols that systematically integrate manual therapy, dry needling, therapeutic exercise, and pain education to optimize the clinical management of TTH. Interestingly, the progressive, staged approach reported by physiotherapists (starting with suboccipital and upper cervical techniques, followed by interventions targeting the trapezius, SCM, masticatory muscles, and later incorporating therapeutic exercise) could be interpreted as a potential framework for implementing evidence-informed approaches in real-world settings, while formal consensus-based guidelines are being developed.

### 4.2. Clinical Implications

The findings suggest several potential implications for clinical practice, as suggested by the survey responses. First, the suboccipital and upper cervical region may be considered a priority therapeutic target, due to its repeated implication in TTH [30,31,32]. In addition, combining specific and global techniques may optimise clinical effectiveness, endorsing a multimodal approach [53]. Evaluation of the cranio-cervico-mandibular complex may also be warranted, given the trigeminocervical relationships reported in the study [11,46]. Therapeutic exercise could assume a more prominent and earlier role, particularly for chronic or recurrent presentations [54]. Similarly, the incorporation of biopsychosocial strategies appears advisable, including pain education, stress management and self-management strategies [43,44]. In the Spanish context, the widespread use of dry needling alongside the limited incorporation of pain education suggests specific areas where professional training and regulatory guidance could be strengthened. Structured educational programs on safe and effective dry needling techniques, as well as mandatory inclusion of pain neuroscience and biopsychosocial approaches in continuing professional development, could help optimize clinical outcomes and ensure safe, evidence-informed practice. Finally, the variability observed in reported practice highlights the potential value of establishing consensus-based protocols regarding dosage, frequency and evidence-supported combinations.

### 4.3. Limitations

This study presents several limitations that should be taken into account when interpreting the findings. First, convenience sampling may have preferentially captured physiotherapists with particular interest or training in TTH. Second, the survey design describes reported practices but does not allow direct assessment of actual treatment effectiveness. Similarly, some responses were excluded due to insufficient specificity, which could affect the representation of certain modalities. In addition, the observed variability in responses may be influenced by differences in professional profiles, clinical decision-making criteria and patient characteristics.

Finally, as the data are based on self-reported responses, they may not fully reflect actual clinical behavior and are subject to recall bias and social desirability bias. Additionally, the recruitment strategy did not allow calculation of a response rate, and the use of non-probabilistic sampling limits the representativeness and generalizability of the findings. Furthermore, the questionnaire was not formally psychometrically validated, which may limit the interpretability of responses. Recruitment through professional networks may also have biased the sample toward physiotherapists with greater expertise or interest in TTH management, further constraining generalizability.

### 4.4. Future Research Directions

Based on these findings, several potential needs can be identified for future research. There is an apparent need for controlled clinical trials to further evaluate the effectiveness of the most commonly used protocols, preferably employing high methodological standards. Likewise, the development of consensus-based clinical guidelines defining techniques, dosage, sequencing and frequency in TTH management could be valuable. Qualitative studies exploring how physiotherapists make clinical decisions, the barriers they encounter, and their perceptions of therapeutic effectiveness may also provide important insights. Finally, research on multimodal treatments assessing their potential impact on quality of life, function, medication use and healthcare costs would be highly informative.

## 5. Conclusions

This survey provides an exploratory overview of the range of techniques (manual therapy, exercise, invasive procedures, electrotherapy, and complementary approaches) used by physiotherapists in the management of tension-type headache (TTH). Many of these reported practices, particularly manual therapy and exercise, appear to align with evidence-based recommendations. However, the considerable heterogeneity in technique selection, target structures, treatment dosage, and integration of biopsychosocial strategies may reflect gaps between real-world practice and standardized protocols reported in recent scoping reviews. In particular, the reported use of dry needling and the limited incorporation of pain education may highlight areas where further guidance and standardization could be beneficial. Overall, these findings should be interpreted as exploratory and suggest the need for future research and the development of clearer guidelines, training frameworks, and integration of biopsychosocial strategies to support clinical practice.

## Figures and Tables

**Table 1:** Thematic structure of the final questionnaire, including the distribution of closed- and open-ended questions across five sections.

Sociodemographic and Professional Information	Gender	Age	Level of Education	–	“Age”
Experience with Patients with TTH	Years of experience	Professional setting as a physiotherapist	Patients with TTH treated per month	–	“In which setting do you primarily practice as a physiotherapist?”
Treatment Practices	Treatment modality commonly used to manage TTH	MTT considered most useful for TTH	IT considered most useful for TTH	ET considered most useful for TTH	“What treatment modality do you usually use to manage TTH?”
Perceptions of prioritized therapeutic techniques	First-line technique and target area	Second-line technique and area of application	Tertiary technique and area of application	–	“If you had to standardize the physiotherapeutic management of TTH using only three specific techniques, which would you prioritize?”
Intervention protocols	Number of sessions per patient	Frequency of sessions	Duration of each session	Use of structured protocol vs. individualized approach	“Do you follow a structured protocol or adapt it individually for each patient?”

ET: Electrotherapy techniques; IT: Invasive therapies; MTT: Manual therapy techniques; TTH: Tension-Type Headache.

**Table 2:** Sociodemographic and Professional Characteristics of the Sample.

Variable	Category/Statistic	%	Description/Value
Gender	Female	50%	—
	Male	50%	—
Age	Mean	—	36.01 years
Academic degree	Bachelor’s degree	40%	—
	Master’s degree	44%	—
	Doctorate	16%	—
Years of experience	0–5 years	28%	—
	5–10 years	23%	—
	>10 years	49%	—
Professional activity	Private clinical practice	66.8%	—
	University teaching	9.3%	—
	Research	10%	—
	Associations/organizations	4.6%	—
	Public institution	9.3%	—
Number of patients treated per month	0–20	73%	—
	20–40	24%	—
	>40	3%	—

## Data Availability

Anonymized data may be available under reasonable request.

## Supplementary Materials

The following supporting information can be downloaded at https://www.mdpi.com/article/10.3390/jcm15082896/s1: Table S1: Distribution of Commonly Used Manual Therapy Techniques; Table S2: Commonly used therapeutic exercise modality; Table S3: Invasive therapies modalities used in clinical practice; Table S4: Commonly used electrotherapy modality; Table S5: Other commonly used therapeutic modalities; Table S6: Preferred treatment modalities and target areas.
