# Supplementary material for: Clinical Practice Patterns in the Physiotherapy Management of Tension-Type Headache Among Spanish Physiotherapists
Source: J Clin Med. 2026 Apr 10;15(8):2896. doi: 10.3390/jcm15082896 (PMC13116893; doi:10.3390/jcm15082896)
Supplement: Supplementary file 1 [file jcm-15-02896-s001.zip › jcm-4209071-supplementary.pdf]

Table S1. Distribution of Commonly Used Manual Therapy Techniques.

Category	Modality	Treated Area	Frequency
Soft Tissue Therapy	Muscle inhibition	Suboccipital	46
		Masticatory	7
		SCM	7
		Trapezius	7
	Massage therapy	Cervical	13
		Orofacial	4
		Thoracic	3
		Masticatory	3
		Suboccipital	1
	Myofascial release	Cervical	7
		Cranial	3
		Orofacial	1
		Tongue	1
	Stretching / relaxation	Cervical and paravertebral muscles	12
	Trigger point treatment	Cervical	4
		SCM	2
		Masticatory	3
Joint Mobilization and Manipulation	Joint mobilization	Cervical (C0–C3)	24
		Thoracic	7
		Cranio-cervical	3
	Spinal manipulation	Cervical	6

Category	Modality	Treated Area	Frequency
		Thoracic	4
		Sacral	1
	Traction / distraction	Upper cervical	7
	Mulligan techniques	Cervical and thoracic	5
TMJ and Orofacial Approach	TMJ manual therapy	Masseter, pterygoids, temporalis	20
	Intraoral techniques	Deep masticatory muscles, tongue	8
Cranial and Craniosacral Treatment	Cranial techniques	Temporals, sphenoid, intracranial membranes	12
	Cranial mobility	Cranium	7
	Craniosacral therapy	Cranial-sacral	6
Osteopathic Techniques	Cranial osteopathy	Cranium	7
	Sphenoidal/ membrane balancing	Sphenoid, intracranial membranas	6
	Global osteophathic manipulation	Spine and sacrum	5
Neuromuscular Techniques and Neurodynamics	Neuromuscular techniques	Temporalis, masseter, cervical muscles	7
	Neurodynamics	Arnold's nerve, trigeminal nerve	3
Deep Fascia and Diaphragm	Deep fascial release	Cervical fascia, diaphragm	4

SCM: sternocleidomastoid; TMJ: temporomandibular joint

Table S2. Commonly used therapeutic exercise modality.

Modality	Treated Area	Frequency
Mobilization / Range of Motion	Cervical	22
	Scapular / shoulder	6
	Thoracic	5
	TMJ / cranio-cervical	5
Strengthening / Endurance	Cervical	15
	Scapular / shoulder	10
	Thoracic	4
	TMJ / cranio-cervical	3
Isometrics / Static contractions	Cervical	19
	Scapular / shoulder	5
Stretching / Flexibility	Cervical	15
	Scapular / shoulder	5
	Thoracic	4
Motor control / Postural re-education	Cervical	20
	Scapular / shoulder	4
Proprioception / Neuromuscular training	Cervical	5
	Scapular / shoulder	2
Aerobic / Cardiovascular exercise	General	7
Breathing / Diaphragmatic exercises	Cervical / thoracic	7

**Modality**	**Treated Area**	**Frequency**
Functional training / Upper body strengthening	Scapular / shoulder	6
Specific TMJ / ocular exercises	TMJ / cranio-cervical	6
Pilates / Yoga / Mind–body	General	4
Neurodynamics / Nerve mobilization	Cervical / arm	3

TMJ: temporomandibular joint

Table S3. Invasive therapies modalities used in clinical practice.

**Modality**	**Treated Areas**	**Frequency**
Dry Needling	Upper, middle, and general trapezius	22
	Suboccipitals	14
	Masseter / masticatory muscles	8
	Levator scapulae	8
	SCM	5
	Splenius / semispinalis muscles	5
PENS	General	12
	Suboccipital	7
	Levator scapulae	1
	Infraspinatus	1
	Trapezius	1
	Arnold’s nerve / greater occipital nerve	9

Modality	Treated Areas	Frequency
	Trigeminal nerve	4
	Neuroaxis / spinal	3
Acupuncture	General / unspecified	1

PENS: percutaneous electrical nerve stimulation; SCM: sternocleidomastoid

Table S4. Commonly used electrotherapy modality.

Modality	Treated Area	Frequency
TENS	Cervical	15
	Trapezius / scapular region	12
	Arnold´s nerve/ occipital/ phrenic/ cervical	9
	SCM / scalenes	4
	TMJ / cranio-cervical	1
Radiofrequency	Cervical/ thoracic/ suboccipital	12
Superinductive therapy	Cervical / thoracic	4
Ultrasound	Cervical / trapezius / TMJ	4
Laser/ Inductive therapy	Cervical/ thoracic	2
Magnetotherapy	General	1
Transcranial electrical stimulation	Head / cranial	1
Microwaves	Thoracic	1
Diamagnetic pump	Cervical / thoracic	1

SCM: sternocleidomastoid; TENS: transcutaneous electrical nerve stimulation; TMJ: temporomandibular joint

Table S5. Other commonly used therapeutic modalities.

Modality	Frequency
Pain education	8
Kinesiology taping (paravertebral, cervical, trapezius)	6
Cupping	1
Cross tapping	1

Table S6. Preferred treatment modalities and target areas.

Technique	Treated Area	1st Choice	2nd Choice	3rd Choice	Total
Inhibition	***Suboccipital***	***18***	***9***	***3***	***30***
	Upper cervical / C0–C3	4	3	2	9
	Temporalis / pterygoids / TMJ	4	3	1	8
	Upper trapezius / levator scapulae	5	4	2	11
Mobilization	***Cervical / cervicodorsal***	***12***	***8***	***3***	***23***
	Suboccipital	5	3	3	11
	TMJ	1	1	1	3
Dry needling	Suboccipital muscles	9	6	5	20
	Temporalis / masseter / TMJ	6	3	2	11
	Trapezius	5	4	2	11
	General cervical region	3	2	2	7
Massage therapy	Cervical / cervicodorsal	6	8	9	23
	Suboccipital region	4	5	6	15
	TMJ / masticatory muscles	1	3	4	8

Technique	Treated Area	1st Choice	2nd Choice	3rd Choice	Total
Myofascial techniques	Suboccipital	6	8	9	23
	Cervical / trapezius / SCM	5	7	7	19
	TMJ / masseter / temporalis	2	4	5	11
Manipulation	Cervical (general)	4	3	3	10
	Upper cervical	3	3	2	8
	Thoracic	1	1	1	3
	Suboccipital	1	1	1	3
Exercise therapy	***Deep cervical stabilizers***	***3***	***6***	***12***	***21***
	Aerobic	1	10	3	14
	Respiratory / diaphragm	0	2	6	8
Osteopathic techniques	Upper cervical / cervicothoracic junction	3	4	2	9
	Cranial / membranes / sphenoid	2	3	2	7
	Visceral / diaphragm	1	1	2	4
Neuromodulation	Arnold's nerve	1	7	4	12
	TMJ / trigeminal	1	5	4	10
	Cervical region	0	4	3	7
Electrotherapy	Cervical / upper thoracic	1	6	6	13
	Cranial / TMJ	0	2	4	6

SCM: sternocleidomastoid; TMJ: temporomandibular joint
